# Three-dimensional free breathing whole heart cardiovascular magnetic resonance T_1_ mapping at 3 T

**DOI:** 10.1186/s12968-018-0487-2

**Published:** 2018-09-17

**Authors:** Rui Guo, Zhensen Chen, Yishi Wang, Daniel A. Herzka, Jianwen Luo, Haiyan Ding

**Affiliations:** 1 Center for Biomedical Imaging Research, Department of Biomedical Engineering, School of Medicine, Tsinghua University, Beijing, China; 2 Department of Biomedical Engineering, Johns Hopkins School of Medicine, Baltimore, MD USA; 3 National Heart, Lung, and Blood Institute, National Institutes of Health, Bethesda, MD USA

**Keywords:** Myocardial T_1_ mapping, Three-dimensional, Free-breathing, Saturation, 3 T, Tissue characterization, Native T_1_, Post-contrast T_1_, Cardiovascular magnetic resonance

## Abstract

**Background:**

This study demonstrates a three-dimensional (3D) free-breathing native myocardial T_1_ mapping sequence at 3 T.

**Methods:**

The proposed sequence acquires three differently T_1_-weighted volumes. The first two volumes receive a saturation pre-pulse with different recovery time. The third volume is acquired without magnetization preparation and after a significant recovery time. Respiratory navigator gating and volume-interleaved acquisition are adopted to mitigate misregistration. The proposed sequence was validated through simulation, phantom experiments and in vivo experiments in 12 healthy adult subjects.

**Results:**

In phantoms, good agreement on T_1_ measurement was achieved between the proposed sequence and the reference inversion recovery spin echo sequence (R^2^ = 0.99). Homogeneous 3D T_1_ maps were obtained from healthy adult subjects, with a T_1_ value of 1476 ± 53 ms and a coefficient of variation (CV) of 6.1 ± 1.4% over the whole left-ventricular myocardium. The averaged septal T_1_ was 1512 ± 60 ms with a CV of 2.1 ± 0.5%.

**Conclusion:**

Free-breathing 3D native T_1_ mapping at 3 T is feasible and may be applicable in myocardial assessment. The proposed 3D T_1_ mapping sequence is suitable for applications in which larger coverage is desired beyond that available with single-shot parametric mapping, or breath-holding is unfeasible.

**Electronic supplementary material:**

The online version of this article (10.1186/s12968-018-0487-2) contains supplementary material, which is available to authorized users.

## Background

In cardiovascular magnetic resonance (CMR), myocardial T_1_ mapping provides a non-invasive tool for direct quantification of changes in tissue characterization. Native T_1_ is sensitive to tissue properties such as water content, fat, iron deposition and fibrosis [1, 2], and has been used to identify a broad variety of cardiomyopathies [3–6]. Additionally, measurement of myocardial T_1_ after infusion of T_1_-shortening contrast agents can be used to estimate extracellular volume (ECV) fraction. This is particularly valuable for detecting diffuse myocardial diseases to which conventional late gadolinium enhancement CMR is insensitive [7]. Both native and post-contrast myocardial T_1_ mapping are important CMR techniques of ever increasing value in clinical diagnosis, prognosis and long-term follow-up of various cardiac diseases [8].

T_1_ mapping techniques generally acquire multiple co-registered images with varying amounts of T_1_ relaxation and quantify T_1_ by fitting with exponential recovery models on a pixel-by-pixel basis. Current techniques, such as modified Look-Locker inversion recovery (MOLLI) [9] and saturation recovery single-shot acquisition SASHA [10], usually adopt a two-dimensional (2D) acquisition. These techniques differ in their T_1_ preparation strategies but most employ breath-hold and single-shot imaging. Breath-holding limits the total scan time available for acquisition of multiple single-shot images as typically one image is acquired per heartbeat. Thus, spatial resolution and coverage are also limited and the imaging is susceptible to lack of patient compliance. Furthermore, 2D imaging is intrinsically subject to through-plane motion artifact [11] and low signal-to-noise ratio (SNR) [10, 12] when using desired thinner slice, both of which can compromise the quality of parametric maps. The accuracy of the estimate of T_1_ may be further degraded by the misregistration of images, which can result from large acquisition window extending beyond diastasis along with poor breath-holding or heart rate variation [13]. Motion correction methods have been developed to improve the map quality. However, these methods generally are limited to in-plane motion and require relatively complex processing [14, 15], and their validity has not been fully demonstrated.

Three-dimensional (3D) acquisition has inherently higher SNR and allows more comprehensive left ventricular (LV) characterization due to its larger coverage, and higher potential in-plane and through-plane resolutions. However, 3D acquisition typically requires longer scan time due to simultaneous cardiac and respiratory motion compensation. This poses a challenge for accurate 3D myocardial T_1_ mapping, especially when measuring the relatively long native T_1_ at high magnetic fields. Several techniques for 3D T_1_ mapping have been proposed [16–22]. Among these techniques, some are designed for post-contrast myocardial T_1_ mapping [18, 20] and difficult to be extended for native myocardial T_1_ mapping. And some use inversion-recovery preparation, making the measured T_1_ sensitive to the variation of heart rate [21, 22]. Another technique using variable flip angle with 2D B_1+_ calibration showed fair quality of T_1_ map due to the variation of B_1+_ field across the left ventricle [16, 23]. At 1.5 T, a 3D implementation of SASHA has addressed these issues by using saturation preparation and acquiring an equilibrium volume [17]. Compared with 2D SASHA [10], 3D SASHA achieves superior image and parametric map quality at the expense of increased scan time.

In this study, we propose a 3D free-breathing saturation-based T_1_ mapping sequence for myocardial tissue characterization at 3 T. In this sequence, the influence of field inhomogeneity at high field was mitigated by using radiofrequency-spoiled gradient echo (SPGR) sequence as readout, and the sensitivity to heart rate variation was reduced by adopting saturation prepulse. Additionally, a volume-interleaved acquisition fashion with respiratory navigation was designed to obtain an accurate measurement of the equilibrium magnetization thus ensuring accuracy of the T_1_ map. The proposed sequence was validated with simulations, phantom and in vivo experiments.

## Methods

All imaging studies were performed on a 3 T CMR System with multi-transmit capability (Achieva TX, Philips Healthcare, Best, Netherlands). Phantom studies used an 8-channel head coil and the in vivo studies used a 32-channel cardiac coil. The human study was approved by the local institutional review board. Written informed consent was obtained from all subjects. Numerical simulations, image processing, and statistical analysis were performed in MATLAB (MathWorks, Natick, Massachusetts, USA).

### Pulse sequence design

**Fig. 1:**
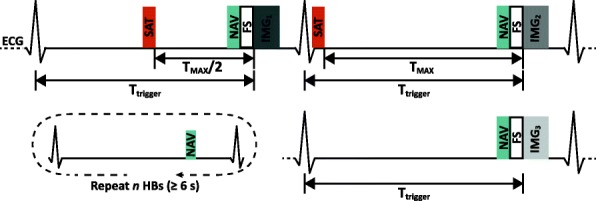
Pulse sequence diagram for the proposed free-breathing 3D T_1_ mapping sequence. Three electrocardiogram (ECG) triggered differently T_1_-weighted volumes (IMG_1_, IMG_2_ and IMG_3_) were acquired in an interleaved fashion. The magnetization for IMG_1_ and IMG_2_ was prepared by a saturation (SAT) pulse with different delay time (T_SAT1_ and T_SAT2_). *n* heartbeats (HBs) were skipped before the equilibrium volume (IMG_3_) was acquired, where *n* was calculated using Eq. A1 to achieve a T_1_ recovery time ≥ 6 s. Respiratory navigator (NAV) gating and fat suppression (FS) were employed. For IMG_3_, after the prescribed *n* HBs, the readout will be performed only if the respiratory navigator is within the acceptance window

The proposed 3D T_1_ mapping technique uses 3 differently T_1_-weighted volumes to generate the pixel-wise T_1_ maps. The sequence (Fig. 1) includes modules of saturation (SAT), respiratory navigator (NAV), fat suppression (FS) and image readout. The volumes are acquired using a multi-shot SPGR sequence in an interleaved fashion during diastole. The SAT-prepared volumes, IMG_2_ and IMG_1_, utilize different delay times (T_SAT_) corresponding to the maximal available delay time (T_MAX_) allowed by the system and half of T_MAX_ (T_MAX_/2), respectively. IMG_3_ is acquired several heartbeats after the IMG_2_, without SAT preparation, to obtain the fully recovered equilibrium magnetization. Hence, a delay time of ≥6 s is desired for native T_1_ mapping (see the simulation below), and the number of T_1_ recovery heartbeats needed is calculated and updated in real time before each shot (Appendix). Despite heart rate fluctuation, the targeted minimum delay time is always achieved.

SAT was implemented by using the WET (water suppression enhanced through T_1_ effects) technique [24]. For in vivo studies, FS was applied right before the readout using spectral presaturation with inversion recovery (SPIR). Free-breathing acquisitions with respiratory motion compensation were achieved with a pencil-beam NAV on the dome of the right hemidiaphragm [25]. The NAV data were acquired before the readout.

### T_1_ calculation

The pixel-wise T_1_ map was calculated from the differently T_1_-weighted volumes using nonlinear least squares algorithm, with the following signal evolution model:

1 $$ S=\mathrm{A}-\mathrm{A}\ast \exp \left(-\frac{T_{SAT}}{{\mathrm{T}}_1}\right) $$

Where A is the signal with full recovery of the longitudinal magnetization, *S* is the measured signal intensity, and *T*_*SAT*_ is the saturation recovery duration. We assume that for a given voxel, the magnetization at the onset of imaging was a function of T_1_ and *T*_*SAT*_.

### Simulations

The T_1_ recovery durations would affect the image signal intensities, and thus accuracy and precision of the T_1_ measured by the proposed sequence. These quantities are all dependent on heart rate. In this study, the following aspects were simulated: a) the available magnetization of IMG_1_ and IMG_2_ under various heart rates; b) the magnetization of IMG_3_ (i.e. the equilibrium volume) under different recovery time. Since the longitudinal magnetization is very small after the readout of IMG_2_ due to the use of SPGR, the initial signal intensity in this simulation was assumed to be zero; c) the relative error of T_1_ estimation induced by imperfect T_1_ recovery of IMG_3_.

A full recovery of the equilibrium magnetization before the acquisition of IMG_3_ is desired for an accurate T_1_ estimation but it comes at the expense of scan efficiency. Due to the periodic nature of the cardiac cycle, the T_1_ recovery period for IMG_3_ can only be specified by an integer number of heartbeats. Variations in heart rate during T_1_ recovery eventually can result in degraded T_1_ estimation, as adopting an infinite or very long recovery time is impractical. To explore this heart rate dependency, Monte Carlo simulation was performed. The single simulation was repeated 10,000 times, with the heart rate varying with a prescribed mean value and a standard deviation (SD) of 5 bpm. The number of the idle heartbeats before imaging IMG_3_ was calculated with respect to the prescribed mean heart rate (Appendix) to achieve a T_1_ recovery duration ≥6 s.

In the above simulation, the magnetization as a function of the T_1_ was calculated using Bloch equations. The simulated T_1_ ranged from 500 ms to 1750 ms, and the heart rate from 40 bpm to 120 bpm. The efficiency of the SAT was assumed to be 100%. T_MAX_ was calculated as 80% of the R-R interval. T_SAT_ of IMG_1_ and IMG_2_ were set to T_MAX_/2 and T_MAX_.

### Phantom studies

Phantom experiments were performed to validate the proposed sequence. Twelve gel phantoms, of which the T_1_ and T_2_ values span the possible myocardial relaxation times at 3 T (T_1_: 500–1800 ms, T_2_: 30–80 ms) [26], were prepared using different concentration of agarose (Sigma-Aldrich, Saint Louis, Missouri, USA) and gadopentetate dimeglumine (Gd-DTPA, Magnevist, Bayer Pharma AG, Germany).

To evaluate the accuracy of the T_1_ measured by the proposed sequence, all phantoms were scanned with both the proposed sequence and an inversion-recovery spin-echo (IR-SE) sequence (taken as reference in this study). Imaging parameters of the proposed sequence were: flip angle (FA) 18° (see Additional file 1: Figure S1 for the rationale of this FA value), TR/TE 2.28/0.78 ms, partial echo factor 0.625, field-of-view (FOV) 150 × 150 × 30 mm^3^, voxel size 2 × 2 × 10 mm^3^, and acquisition window 76 ms (i.e. 33 readouts per heartbeat) at a simulated heart rate of 60 bpm. T_SAT_ of IMG_1_ and IMG_2_ were 395 ms and 788 ms. The targeted minimum recovery time for IMG_3_ was set to 6 s, the same as for the in vivo experiments. Neither FS nor NAV were used. Imaging parameters of the IR-SE sequence were: 14 inversion times (100–3000 ms), FOV 150 × 150 mm^2^, voxel size 2 × 2 × 10 mm^3^, TR 10 s. The reference T_1_ maps from the IR-SE sequence were calculated using the following three-parameter model:

2 $$ S=\mathrm{A}+\mathrm{B}\ast \exp \left(-\frac{T_{INV}}{{\mathrm{T}}_1}\right) $$

where *S* is the measured signal intensity, *T*_*INV*_ is the T_1_ recovery duration after the inversion pulse, A, B and T_1_ are the unknowns.

To explore the sensitivity of the proposed sequence to the heart rate, another nine scans of the proposed sequence were performed with the simulated heart rate changing from 40 bpm to 120 bpm at a step size of 10 bpm. T_SAT_ of IMG_1_ and IMG_2_ were set to T_MAX_/2 and T_MAX_, which would change along with the heart rate. The targeted minimum recovery time for IMG_3_ was 6 s as well.

The saturation efficiency (*η*) of the WET pulse on the phantoms was experimentally measured using a gradient echo sequence with the following parameters: FA 15°, TE 2.1 ms, FOV 150 × 150 mm^2^, voxel size 2 × 2 × 10 mm^3^ and TR 15 s. Seven images were acquired: one without saturation preparation, and the others six prepared by a saturation pulse with delay times ranging from 300 ms to 1200 ms [10]. The *η* was calculated using the following model:

3 $$ S=\mathrm{A}\left(1-\eta \ast \exp \left(-\frac{T_{SAT}}{{\mathrm{T}}_1}\right)\right) $$

where, A, *η* and T_1_ were the unknowns.

### In-vivo studies

Twelve human adult subjects (7 males, 30 ± 12 yrs) without a history of heart disease were recruited. Each subject was scanned with the proposed sequence and 2D SASHA. The imaging parameters for the proposed sequence were: electrocardiogram (ECG)-triggered multi-shot SPGR, partial echo factor 0.625, FOV 280 × 300 × 96 mm^3^, voxel size 1.5 × 1.5 × 16 mm^3^, which was reconstructed to 1.5 × 1.5 × 8 mm^3^, FA 18°, TR/TE 3.3/1.0 ms, respiratory navigator acceptance window 5 mm. The acquisition window was kept to 100–115 ms (i.e. 31–35 k-space lines was acquired per shot) to minimize the effect of cardiac motion and reduce partial volume averaging. T_SAT_ for IMG_1_ and IMG_2_ were ~ 300 ms and ~ 600 ms, respectively, depending on the heart rate. No parallel imaging was used. Images were reconstructed using a dual-phase partial echo reconstruction method [27]. 2D SASHA completed in a single breath-hold was performed at the mid-LV short-axis-view (SAX) level with the parameters: single-shot ECG-triggered balanced steady-state free precession (bSSFP) with 10 start-up echoes, FOV 280 × 300 × 10 mm^3^, voxel size 1.7 × 2.1 × 10 mm^3^, FA 35°, TR/TE 3.0/1.3 ms, sensitivity encoding (SENSE) acceleration rate 2, partial Fourier factor 0.875, T_SAT_ ranging from 100 ms to 700 ms. Volumetric first-order B_0_ shimming and volumetric B_1+_ shimming were performed to compensate the field inhomogeneity before the T_1_ mapping scans [28]. The shimming volume was manually defined carefully to include the heart only.

In addition, the applicability of the proposed sequence for post-contrast T_1_ mapping was preliminarily studied on another two human subjects (males, age 46 ± 6 yrs). In this experiment, the proposed sequence used a targeted minimum recovery time of 3 s for IMG_3_, and was performed 15 min after injection of Gd-DTPA at 0.15 mmol/kg (Magnevist, Bayer Pharma AG, Germany).

### Image analysis

#### Phantom experiments

A center-aligned circular region of interest (ROI) was drawn on each phantom on the reference T_1_ maps and transferred onto the T_1_ maps obtained by the proposed sequence as well as the saturation efficiency map for calculating the mean and SD of T_1_ or *η*. The correlation between the T_1_ values from the proposed sequence and from IR-SE sequence was calculated by using linear regression. Bland-Altman analysis was also performed between the two sequences. The relative error (i.e. percentage estimation error relative to the T_1_ value from the IR-SE sequence) and the coefficient of variation (CV, the SD divided by the mean) were calculated for evaluation of the accuracy and precision of T_1_ measured by the proposed sequence.

### In-vivo Experiments

T_1_ maps from 2D SASHA were calculated using both two-parameter (2-Param) and three-parameter (3-Param) models [13]. No registration among T_1_-weighted volumes or images was performed before T_1_ calculation.

The LV was manually segmented slice by slice on the T_1_ maps from the proposed sequence by consensus between two observers. The long axis view was generated by reformatting the 3D dataset. To perform a comparison with 2D SASHA, the most position-matched slice was extracted from all the slices acquired by the proposed sequence. The mean and SD of the T_1_ values within the septal ROIs were calculated for both the proposed sequence and 2D SASHA. Then the T_1_ values of the two sequences were compared using a paired two-tailed Student’s *t*-test with significance level of 0.05.

All SAX slices of the proposed sequence were evenly categorized into basal, mid-ventricular and apical slabs. Each slice in the base and middle slabs was divided into six segments, while each slice in the apex slab was divided into four segments, according to the American Heart Association (AHA) 17-segment model [29]. The homogeneity and distribution of T_1_ were further evaluated on each segment by measuring the mean, SD and CV. The averaged results of all segments from all subjects were visualized with Bull’s-eye plots to assess the distribution of T_1_ throughout the LV.

## Results

### Simulations

**Fig. 2:**
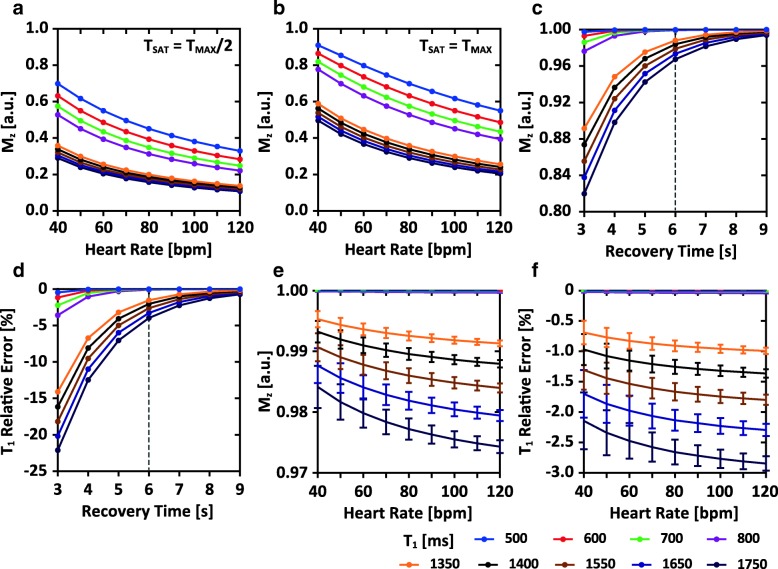
Simulation results of the signal intensity and the accuracy of T_1_ measurement of the proposed sequence. **a** and **b**: the available magnetization for IMG_1_ (**a**) and IMG_2_ (**b**) under different heart rates. The delay time (T_SAT_) of IMG_1_ and IMG_2_ were set to T_MAX_/2 and T_MAX_ respectively, where T_MAX_ was defined as 80% of R-R interval. **c**: the available magnetization for IMG_3_. In this simulation, the initial magnetization was assumed to be 0. **d**: The relative error of T_1_ estimation under the different recovery time of IMG_3_. **e** and **f**: Monte Carlo simulation results of the available magnetization for IMG_3_ (**e**) and the T_1_ relative error (**f**) under different mean heart rates. The targeted minimum recovery time of IMG_3_ was set to 6 s, and the number of idle heartbeats was calculated by Eq. [A1] according to the mean heart rate. The true heart rate during the simulation fluctuated with a standard deviation of 5 bpm during the recovery period

As expected, the heart rate had a strong influence on the available signal especially for long T_1_ species (Fig. 2). Figure 2a and b display the simulated available magnetization for volumes acquired with a T_MAX_/2 and a T_MAX_ saturation-recovery duration, respectively. T_MAX_ was shortened when the heart rate increases, leading to the reduction of the available signal.

Figure 2c and d show the simulated magnetization of IMG_3_ and the resulting relative error of T_1_ over different recovery time. The magnetization of all simulated T_1_ species recovered to > 96% when the recovery duration was > 6 s. For a T_1_ value about 1550 ms, the magnetization recovery achieved about 98% and the corresponding relative error was < 4%.

Figure 2e and f show the magnetization of IMG_3_ and the resulting T_1_ relative error in the Monte Carlo simulation. A relative error < 3% could be achieved for all T_1_ species (Fig. 2e). The typical T_1_ of healthy myocardium (1550 ms at 3 T) [26] could be measured with a relative error < 2.0% even at the highest simulated heart rate (120 bpm). The SD of the relative error was < 0.5% for all T_1_ species when the heart rate presented a 5 bpm fluctuation during the recovery period.

### Phantom studies

**Fig. 3:**
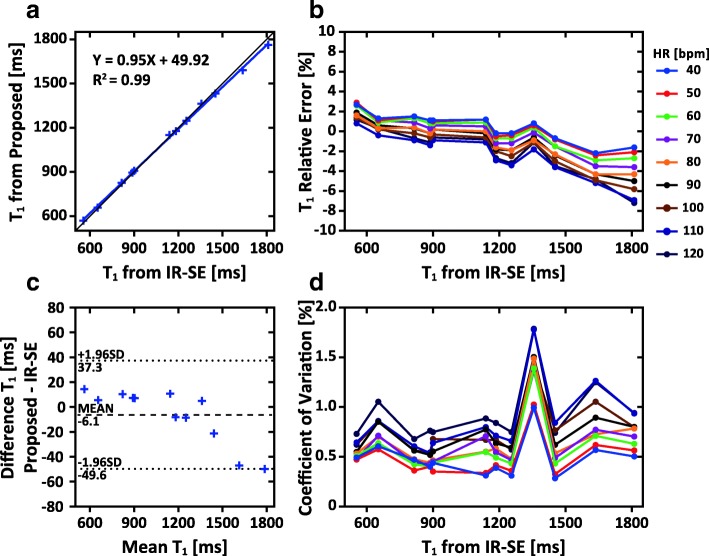
Phantom results of the proposed sequence referring to the inversion recovery spin-echo (IR-SE) sequence. **a**: The correlation between the T_1_ values measured using the IR-SE sequence and the proposed sequence at a heart rate (HR) of 60 bpm. **b**: The relative error of T_1_ measured by the proposed sequence under different heart rates, as compared to the IR-SE sequence. **c**: The Bland-Altman analysis between the proposed sequence and the IR-SE sequence. The dotted line shows the 95% confidence intervals on the limits of agreement. **d**: The coefficient of variation of T_1_ from the proposed sequence under different heart rates

The phantom T_1_ measured by the IR-SE sequence ranged between 524 ms and 1819 ms. Excellent correlation (*R*^*2*^ = 0.99) between the T_1_ values measured by the proposed sequence and the IR-SE sequence was obtained with a small residual and a regression slope of 0.95 (Fig. 3a). The bias in T_1_ between the proposed sequence and the IR-SE sequence was − 6.1 ms with the overall difference < 50 ms (Fig. 3c). The relative error of T_1_ measured by the proposed sequence was in the range of − 7.2 - 2.9% (− 0.9 ± 2.1%) (Fig. 3b), while the CV of T_1_ fluctuated in the range of 0.3–1.8% (0.7 ± 0.3%) (Fig. 3d), when the simulated heart rate ranged from 40 bpm to 120 bpm. The CV obtained from the proposed sequence was not significantly different from that of the IR-SE sequence, under a heart rate of 60 bpm (0.59% vs 0.52%, *p* = 0.35). The measured saturation efficiency of WET pulse was 0.999 ± 0.012 over all phantoms (Additional file 1: Figure S2).

### In vivo studies

The proposed sequence and 2D SASHA were successfully completed on all subjects. The respiratory gating efficiency was between 22 and 60% (39 ± 11%). The averaged scan time was 6.0 ± 1.1 min including the respiratory gating efficiency. The gating efficiency of navigator for IMG_1_ and IMG_2_ is provided in Additional file 1: Table S1. The averaged T_1_ recovery time for IMG_3_ was between 7.0 s and 8.0 s (7.5 ± 0.4 s). Characteristics of the healthy subjects’, HR, recovery time of IMG_3_, mean navigator efficiency and scan time are summarized in Table 1. All subjects were included in the quantitative analysis.

**Table 1:** Characteristics of subjects and the recovery time of IMG_3_ in the in vivo experiments

Subject	Gender	Age	HR	Recovery time of IMG_3_			Navigator efficiency	Scan time
				Max	Min	Mean		
#	(F/M)	(yrs)	(bpm)	(s)	(s)	(s)	(%)	(min)
1	M	51	60.4	17.3	6.3	8.0	33.2	7.5
2	F	22	64.0	10.0	6.1	7.7	45.5	4.6
3	M	62	70.6	11.9	6.4	7.9	34.3	6.3
4	M	24	72.2	14.6	6.4	7.9	22.5	6.9
5	M	27	76.8	9.06	6.1	7.1	57.9	4.2
6	F	25	88.2	8.6	6.1	6.9	32.2	6
7	M	22	74.1	10.7	6.1	7.6	31.4	7.3
8	F	27	53.8	9.8	6.2	7.0	48.9	6
9	M	24	55.0	9.9	6.2	7.4	40.5	6.5
10	F	23	58.9	9.0	6.7	7.6	38	4.9
11	F	23	63.7	9.3	6.1	7.1	29	7.5
12	M	25	69.5	9.5	6.0	7.1	60.6	4.7
μ ± σ		30 ± 12				7.5 ± 0.4	39.5 ± 11.2	6 ± 1.1

*F* female, *M* male, *s* second, *min* minutes, *μ* mean, *σ* standard deviation, *HR* heart rate, *yrs* years, *bpm* beats per minute

**Fig. 4:**
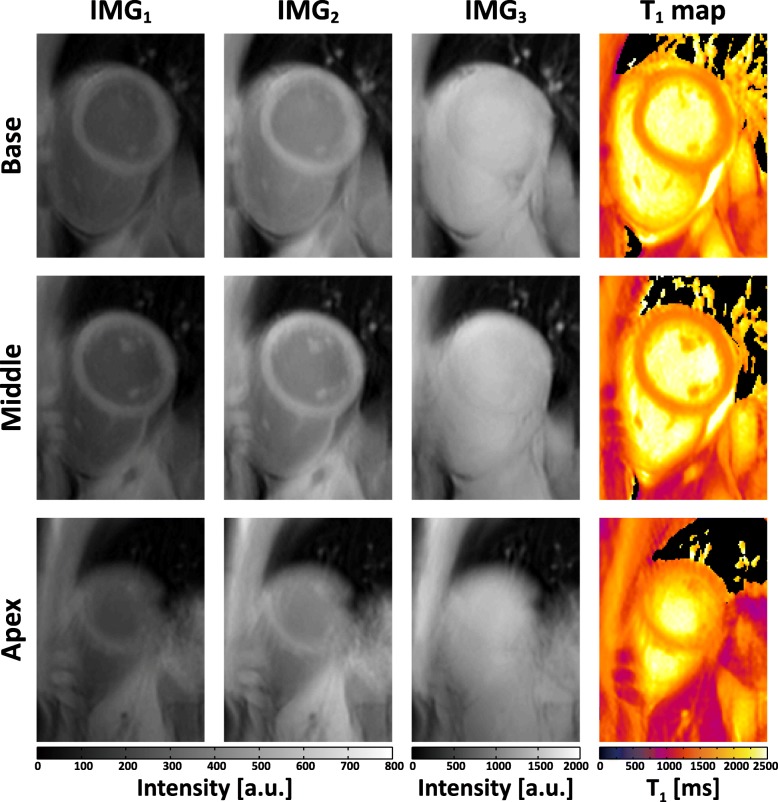
Representative raw weighted images and the corresponding T_1_ maps from the three slabs (i.e. base, middle, apex) of a subject, as obtained by the proposed sequence

**Fig. 5:**
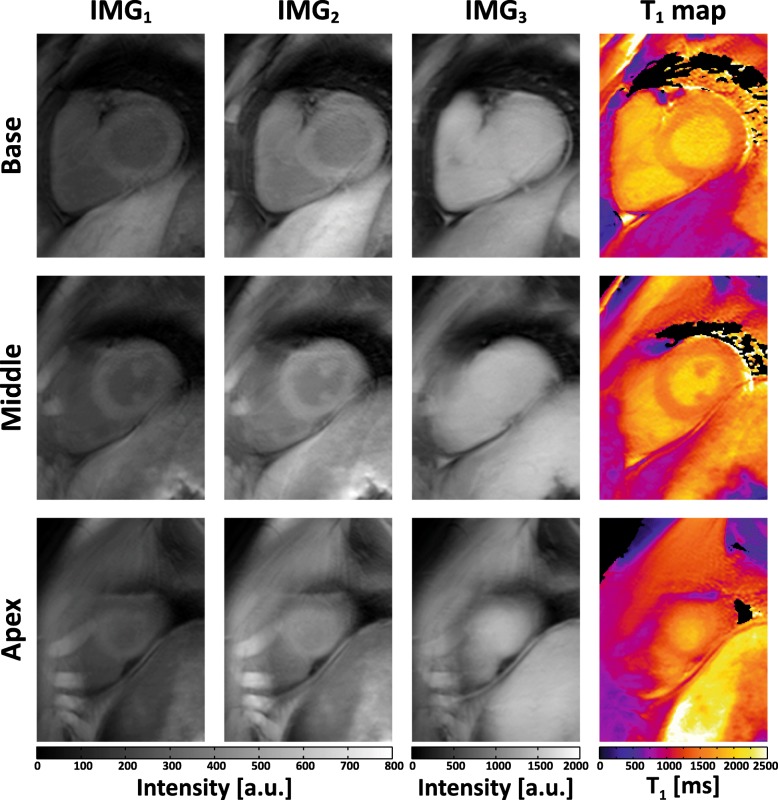
The raw weighted images and the corresponding T_1_ maps from the three heart slabs (i.e., base, middle, apex) of the subject with the most distinct image blurring, as obtained by the proposed sequence

**Fig. 6:**
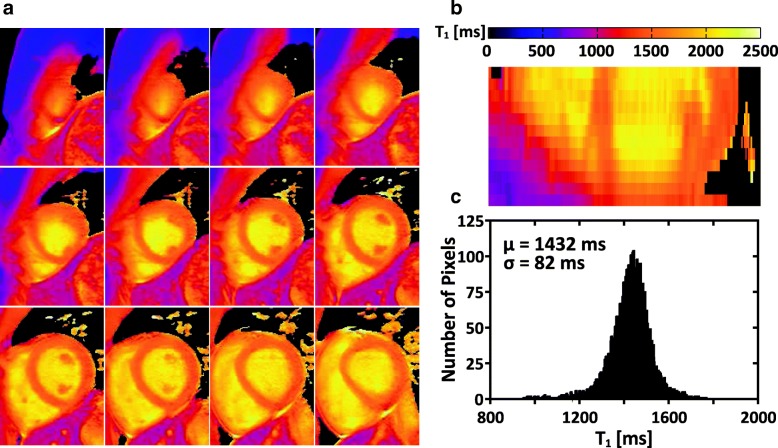
Representative 3D T_1_ maps (**a**) and the corresponding reformatted long-axis view (**b**) from the 3D dataset of one healthy volunteer. **c**: Histogram of myocardium T_1_ over the whole left ventricle. The mean (μ) and standard deviation (σ) of T_1_ values across the whole left ventricle are shown on the graph

**Fig. 7:**
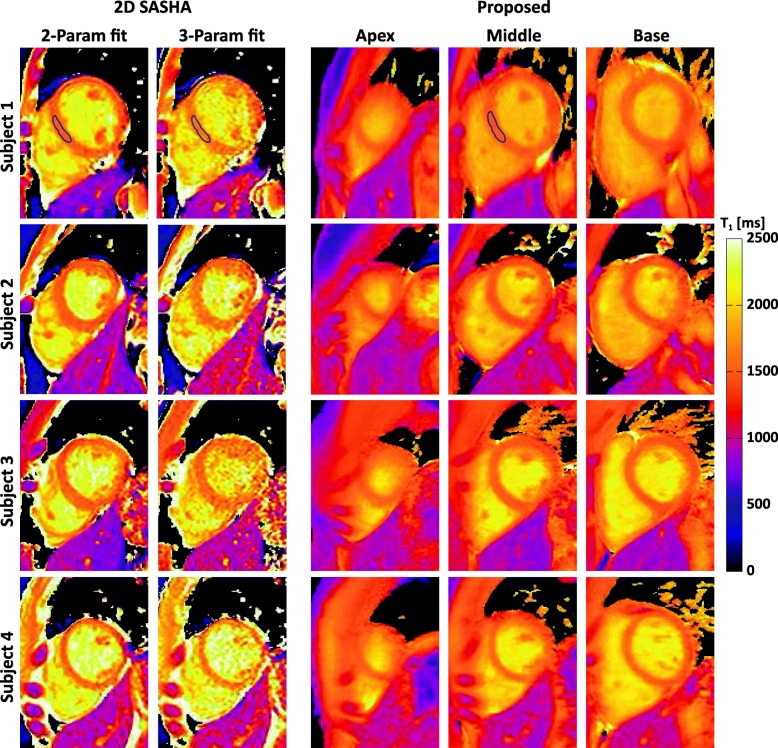
T_1_ maps from 2D SASHA with 2-Param and 3-Param fit, and the proposed sequence with 2-Param fit. The blue ROIs in the first row, which were drawn manually, exemplifies the septal regions whose T_1_ values were used for statistical comparison

Figure 4 shows the raw weighted images acquired by the proposed sequence and the corresponding T_1_ maps from three slabs (i.e. base, middle, apex) of a subject. To illustrate the blurring effect that may present in the proposed sequence due to multi-shot 3D acquisition, images of the subject with the most distinct blurring are shown in Fig. 5. Figure 6 shows representative whole heart 3D T_1_ maps obtained by the proposed sequence. As can be seen, the myocardium T_1_ was homogeneously distributed over the whole LV. The myocardium T_1_ over the whole LV from all subjects was 1476 ± 53 ms, and the CV was 6.1 ± 1.4%. Figure 7 demonstrates the SAX T_1_ maps obtained by the proposed sequence and 2D SASHA with both 2-Param and 3-Param fitting on four subjects.

**Fig. 8:**
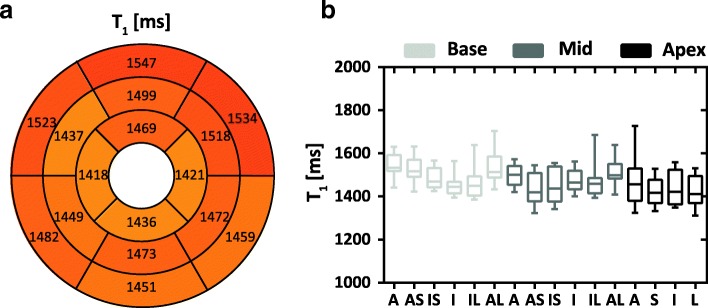
Distribution of myocardium T_1_ over the whole left ventricle, obtained from all volunteers by the proposed sequence. **a.** AHA bull’s eye plot of the mean T_1_ values. **b**. Box and whisker plot of myocardium T_1_ with median, 25 and 75 percentiles, and range. A: anterior; AS: anteroseptal; IS: inferoseptal; I: inferior; IL: inferolateral; AL: anterolateral; S: septal; L: lateral

Figure 8 shows the bull’s-eye and box plots of the myocardium T_1_ based on the AHA 17-segment model. The measured myocardium T_1_ of the apex, middle and base slab were: 1436 ± 71 ms, 1475 ± 52 ms and 1500 ± 49 ms, respectively. Statistically significant statistical difference was found between every two slabs (*p* < 0.01 for all paired t-tests).

**Fig. 9:**
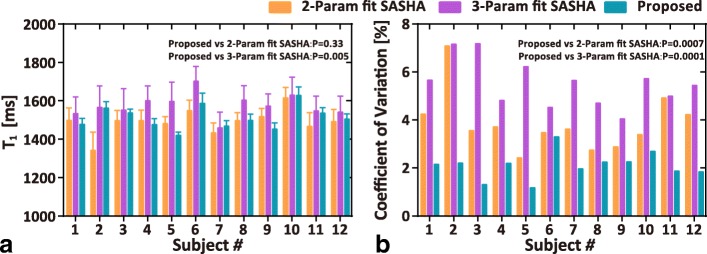
The mean and standard deviation (**a**), and coefficient of variation (**b**) of septal T_1_ measured by 2D SASHA and the proposed sequence. T_1_ of 2D SASHA was calculated using both 2-Param and 3-Param model, while the T_1_ of the proposed sequence was calculated with 2-Param model. Results of paired *t*-test between the proposed sequence and SASHA for the comparison of septal T_1_ and CV are shown within the graph

Figure 9 shows the comparison of the septal T_1_ and CV between 2D SASHA and the proposed sequence. The septal T_1_ from the proposed sequence with 2-Param fit was 1512 ± 60 ms, which was comparable with that from the 2-Param fit SASHA (1490 ± 65 ms) (*p* = 0.33) but lower than that from the 3-Param fit SASHA (1575 ± 59 ms) (*p* = 0.005). The proposed sequence showed much lower CV (2.1 ± 0.5%) than 2D SASHA (3.9 ± 1.2% for 2-Param fit, *p* = 0.0007; and 5.5 ± 0.9% for 3-Param fit, *p* = 0.0001).

The native T_1_ values from the proposed sequence and 2D SASHA on the whole heart myocardium, septum and blood pool are summarized in Table 2.

**Table 2:** The myocardium and blood T_1_ of healthy volunteers (*N* = 12) at 3 T measured by the proposed sequence and 2D SASHA

	2D SASHA		Proposed
	2-Param fit	3-Param fit	
LV T_1_ (ms) (μ ± σ)	–	–	1476 ± 53
LV CV (%)	–	–	6.1 ± 1.4
Septal T_1_ (ms) (μ ± σ)	1490 ± 65	1575 ± 59	1512 ± 60
Septal CV (%)	3.9 ± 1.2	5.5 ± 0.9	2.1 ± 0.5
Blood T_1_ (ms) (μ ± σ)	2281 ± 232	2213 ± 281	2113 ± 142

A mean and CV of T_1_ was calculated for each subject, then the μ, σ, CV and CV’s standard deviation as shown in the table was calculated by across all subjects

*LV* left ventricle, *μ* mean, *σ* standard deviation, *(−)* not applicable, *CV* coefficient of variation

**Fig. 10:**
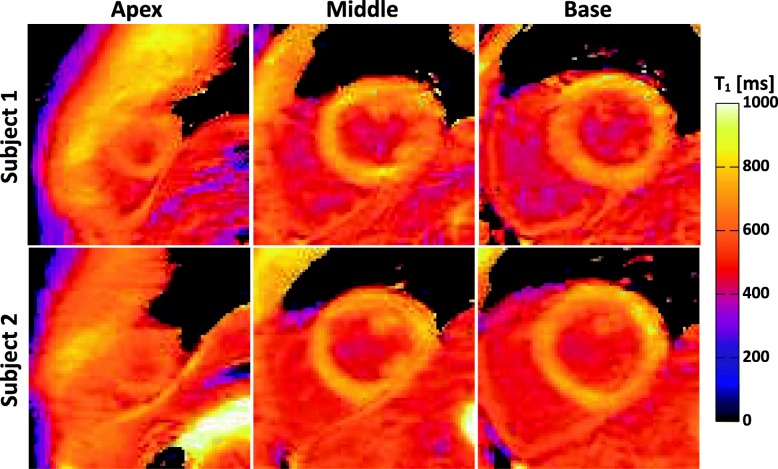
Post-contrast T_1_ maps obtained by the proposed sequence on two healthy subjects. The septal T_1_ at the middle heart slab of the two subjects were 712 ± 28 ms and 731 ± 19 ms, respectively. The blood T_1_ were 438 ± 12 ms and 450 ± 15 ms, respectively

In the additional experiments with contrast injection, homogeneous myocardial T_1_ maps were obtained by the proposed sequence, as shown in Fig. 10 in which a dedicated colormap was used [26]. The measured septal T_1_ at the middle heart slab of the two volunteers were 712 ± 28 ms and 731 ± 19 ms, respectively, while the blood T_1_ were 438 ± 12 ms and 450 ± 15 ms, respectively. Both the post-contrast myocardium and blood T_1_ values conform to the range reported previously [26].

## Discussion

In this study, we demonstrate a 3D free-breathing T_1_ mapping sequence for myocardial T_1_ measurement at 3 T. Whole-heart pixel-wise T_1_ maps were obtained from three co-registered volumes with different T_1_ weightings. The phantom results show that the proposed sequence achieved good accuracy in T_1_ measurement as compared to the reference IR-SE sequence. This technique also features heart rate insensitivity. Whole-heart T_1_ maps with low CV were obtained in the in vivo experiments.

As far as we know, this is the first study that implements a 3D saturation-based sequence for native myocardial T_1_ mapping at 3 T. It is much more challenging to achieve accurate and precise heart T_1_ measurement at high fields such as 3 T, given the prolonged myocardial T_1_ and increased field inhomogeneity [13]. In this study, several tools were used to address these challenges: 1) A composite SAT pulse [24, 30] that was relatively insensitive to the B_1+_ field inhomogeneity was used; 2) A dual-source parallel radiofrequency transmission was used to improve the B_1+_ field homogeneity [28]; 3) SPGR instead of bSSFP was used to avoid the banding artifacts caused by the B_0_ field inhomogeneity; 4) A partial echo readout was used to reduce the susceptibility-induced signal loss; and 5) Both B_1+_ and B_0_ shimming were carefully carried out before the acquisition.

In this study, the respiratory motion was compensated by using the respiratory NAV. Alternatively, breath-holding that is commonly used in 2D cardiac parametric mapping is also feasible for the proposed sequence, although multiple breathholds are required. However, the multiple breathholds fashion would suffer from inter-breath-hold shifting or variability in the breath-hold position, which can lead to motion artifacts and distortion of the T_1_ maps [11]. The motion compensation with respiratory NAV is generally less affected by these effects. The other advantage of the respiratory NAV as compared to bread-holding is that it does not impose limits on the image resolution and coverage [22, 31]. Moreover, free-breathing scans are also more acceptable for elder people and cardiac patients who have difficulty in breath-holding.

Another adaption of the proposed sequence for 3D imaging is that multi-shot data acquisition was employed. In general, multi-shot acquisition allows reduction of the acquisition window, thus minimizing blurring due to cardiac motion and partial volume averaging, an important factor for increasing the sensitivity of T_1_ mapping to disease processes with heterogeneous distribution [13]. More importantly, in this study, interleaved multi-shot acquisition of all T_1_-weighted volumes was used. This led to intrinsic co-registrations of the acquired volumes, and the physiological changes during the scan could be evenly spread over all volumes so that no extra motion correction is needed before the pixel-wise fitting [18, 20, 31].

It has been recommended to acquire a non-SAT image and multiple measurements for the saturation-recovery based T_1_ mapping sequence [32, 33]. This presents a challenge for 3D acquisitions as the only way to acquire data for the non-SAT image is to allow for complete or near-complete relaxation spanning multiple heartbeats. To achieve appropriate relaxation, the prescribed number of idle heartbeats was adapted per shot of the non-SAT volume based on the real-time heart rate to ensure a recovery time of ≥6 s. Though the actual recovery time varied among shots due to the rejection of data by respiratory gating, the minimal 6 s recovery time was always achieved and ensured at least 98% recovery for normal myocardial (~T_1_ 1550 ms) at 3 T [26, 34]. On average, the recovery time (7.5 ± 0.4 s) was longer than the prescribed value in vivo and yielded > 99% recovery ultimately resulting in a high SNR anchor measurement and stable T_1_ fitting with low CV.

The proposed sequence was, by design, robust against the heart rate variability. By means of SAT and the acquisition of an image representing the equilibrium magnetization, an accurate sampling of the T_1_ recovery curve was achieved despite the variation in the duration of the cardiac cycle. In the numerical simulations and phantom experiments (Fig. 2f), with 100% gating efficiency, each shot of IMG_3_ was immediately acquired after the prescribed heartbeats, resulting in elevated relative error in the estimate of T_1_ when the heart rate increased and the recovery time decreased (i.e. smaller rounding off error with respect to 6 s according to Eq. A1). For the in vivo experiments, the number of the idle heartbeats was updated in real-time to ensure a recovery time of ≥6 s and may be increased due to the rejection of data by respiratory gating. Hence, the simulations and phantom experiments represent an upper bound on errors of the T_1_ estimation as they did not include the additional adaptations by respiratory gating in vivo.

We used 2D SASHA as a reference in vivo and used a flip angle of 35° due to the constraints of local energy deposition at 3 T [13]. The reduced flip angle decreases the SNR of the weighted images in comparison with the original 2D SASHA that used 70° at 1.5 T, which in turn decreases the precision of T_1_ estimates [13]. In this study, the in-plane cardiac motion was not corrected before pixel-by-pixel fitting. This could have influenced the estimated T_1_ values. Though non-rigid motion correction methods are available for 2D T_1_ mapping, these algorithms have shown poorer results for saturation-recovery T_1_ mapping methods due to low SNR and contrast in the weighted images [26]. Nevertheless, the septal T_1_ obtained by 2D SASHA with both 2-Param (1490 ms) and 3-Param (1575 ms) fit in this study was reasonable, as compared to the values reported in previous studies [26, 34, 35].

In the proposed sequence, FS was applied to suppress the subcutaneous fat, which would degrade the image quality severely as shown in Additional file 1: Figure S3. Previous studies have shown that the measured myocardium T_1_ has complicated relationship with the regional fat fraction and the imaging parameters [36, 37]. For the myocardial T_1_ measured by the proposed sequence, the application of FS may have two impacts. One is that nulling the fat signal would inevitably affect the resultant T_1_ of myocardium, which contains both water and fat [38]. Another is that the magnetization of water in the myocardium may be disturbed by the imperfect fat-selective RF pulse of the SPIR technique due to the field inhomogeneity. Since most of the conventional T_1_ mapping sequences are commonly performed without using FS, the interpretation of the difference of the T_1_ measured by the proposed sequence to that of other T_1_ mapping techniques should be careful.

The measured septal T_1_ from the proposed sequence (1512 ms) was lower than that from 3-Param fit 2D SASHA (1575 ms) which has been demonstrated to have high accuracy on T_1_ estimation [13]. This discrepancy may be explained by the following factors. First, as shown by the results of the simulation, the proposed sequence has a relative error of about − 1% for T_1_ of 1550 ms even when a 7.5 s recovery time was achieved in vivo. The simulation also showed that this relative error could be further reduced by using a longer recovery time for the equilibrium volume, at the expense of a prolonged scan time. Second, although our data shows that the saturation efficiency of the WET pulse was high on phantoms (Additional file 1: Figure S2), this may be less true in vivo due to the increased B_1+_ inhomogeneity [23]. In this study, a 2-Param model was used for T_1_ calculation for the proposed sequence, without taking the saturation efficiency into account. This effect may also contribute to the underestimation of T_1_. A better design of the SAT pulse to further reduce the influence of the inhomogeneity of B_1+_ field [35] or acquiring more T_1_-weighted volumes to enable a 3-Param fitting could improve the accuracy of the T_1_ estimation by the proposed sequence. Last, the application of FS could also contribute to the difference in T_1_ measurement when compared with conventional T_1_ mapping methods without using FS, as discussed above.

The proposed sequence has high precision on T_1_ measurement as demonstrated by both the phantom and in vivo experiments, although limited number of image volumes were acquired. On the phantom the CV of T_1_ obtained by the proposed sequence was not statistically different from that of the reference IR-SE sequence, while in vivo the CV obtained by the proposed sequence was much lower than that of both the 2-Param fit and 3-Param fit 2D SASHA (Fig. 9). These results suggested that the T_1_ measurement by the proposed sequence has high precision. The precision of T_1_ mapping technique is dependent on several factors, including T_1_, SNR, saturation delay, the signal model and the number of measurements [13, 32, 33]. Considering that the proposed sequence in this study acquire only three images for parameter fitting, we think its high precision should be attributed to the high image SNR resulting from 3D imaging and 2-parameter model fitting. In fact, the image SNR of the proposed sequence was generally higher than that of 2D SASHA, as shown in Additional file 1: Figure S4.

Similar to the previous studies [39, 40], fluctuations of T_1_ among the AHA segments were observed (Fig. 8). Due to the difference in factors like field strength, number of slices, slice thickness, fitting model and sample size between the studies, at the moment it is difficult to determine whether an improvement on depicting the AHA pattern can be achieved by the proposed technique [16, 17, 19, 26]. On the other hand, the observed decreased T_1_ from the inferior to the lateral myocardium may be caused by the well-known susceptibility artifacts and the field inhomogeneity at the heart-liver-lung interface [40].

The proposed sequence can be used to achieve post-contrast myocardial T_1_ mapping with higher efficiency than that for native myocardial T_1_ mapping. Generally, the myocardial T_1_ would be reduced to ~ 800 ms at 3 T after 15 min of the contrast injection. The simulation results showed that for such a T_1_ value, a recovery time of 3 s was sufficient for achieving an equivalent magnetization recovery to the pre-contrast scan during the acquisition of the equilibrium volume. Besides, the simulation results (Fig. 2a and b) also demonstrate that the available signal for IMG_1_ and IMG_2_ in the post-contrast T_1_ mapping will be much higher than that in native T_1_ mapping, which should lead to increased precision. In the present study, homogeneous post-contrast T_1_ maps with the same voxel size as the native T_1_ maps were obtained within 3 min. Performing whole-heart myocardial T_1_ map with and without contrast together could be used to character the regional distribution of ECV and to observe regional heterogeneity.

Though implementing the most comparable sequence 3D SASHA and performing an experimental comparison of the proposed sequence with it is beyond the scope of this work, some information can be given based on a theoretical comparison. The proposed sequence has a similar scheme of signal preparation to 3D SASHA as both rely on SAT [17]. 3D SASHA closely matches 2D SASHA and acquires 9 differently-weighted volumes using bSSFP and has been demonstrated solely at 1.5 T. The equilibrium volume is acquired separately from the other T_1_-weighted volumes with a recovery time of 3 heartbeats. Image resolution of 1.4 × 1.4 × 8 mm^3^ was achieved using SENSE with a 2-fold acceleration in an average scan time of 12 min. The two sequences use different strategies to balance the performance (e.g., accuracy and precision of T_1_) and time efficiency (e.g., the number of the T_1_ weighted images, the use of paralleling imaging) though imaging at 3 T requires additional tradeoffs. At 3 T, energy deposition and B_0_ and B_1+_ fields inhomogeneity preclude the use of 3D bSSFP [41]. Therefore, the proposed sequence used 3D SPGR which is insensitive to field variation. The equilibrium volume was given twice the recovery time for 3D SASHA at the expense of reduced number of weighted volumes. Note that 3D SASHA does benefit from the residual magnetization at the end of a bSSFP readout which is significantly higher than that at the conclusion of the SGPR readout, increasing the effective T_1_ recovery. Additionally, the prolonged myocardial T_1_ at 3 T required a longer recovery time to achieve the equilibrium than that at 1.5 T. Though both techniques acquired the volumes in an interleaved manner [18, 20], 3D SASHA acquired the equilibrium volume separately to better use the residual magnetization of the previous readout. Potential misregistration between the equilibrium volume and the others could happen due to the drift of respiratory state of subject. Clearly, more T_1_-weighted images could improve the precision of T_1_ estimation and parallel imaging, as used in 3D SASHA, could also be applied to the proposed sequence and the increase in efficiency could be used to increase the number of the weighted volumes. However, the effects of parallel imaging on parametric maps (e.g. decrease in SNR, spatially varying noise) are unclear and we opted for full sampling of the data. A more detailed analysis is needed to accurately compare the two techniques and to determine the most efficient sampling strategy.

There are several limitations of this study. First, the long scan time as in our current protocol (~ 6 min) may present a barrier to its clinical application. To reduce the scan time, standard parallel imaging or the current state-of-the-art acceleration strategies such as compressed sensing could be adopted [18], especially given the amount of structural similarity shared among the weighted volumes. The 6 s recovery period needed for an accurate sampling of the equilibrium magnetization is the most time-consuming component in the current approach. This recovery period can be further optimized by a trading off among the precision, fitting models and scan time. Second, interleaved acquisition provides co-registered T_1_-weighted volumes. However, it should be noted that when the volumes are acquired in an interleaved manner, the acquisition time between the first and last k-space segment of each volume is increased, therefore increasing the potential motion artifact that is difficult to predict and correct. Nevertheless, non-interleaved acquisitions would also suffer given that the volumes could represent different physiological states, leading to poor fitting and inaccurate T_1_ estimation.

## Conclusion

We present a 3D free-breathing sequence for myocardial T_1_ mapping at 3 T. The proposed sequence achieves whole heart coverage with low myocardium T_1_ CV. Saturation pulse and long T_1_ recovery duration for the equilibrium acquisition are used to decrease the heart rate dependency. The proposed 3D myocardial T_1_ mapping is feasible for in vivo study at 3 T.

### Additional file

Additional file 1: **Figure S1.** The mean coefficient of variation of phantom T_1_ measured by the proposed sequence under different readout flip angles. **Figure S2.** Results of the phantom experiment for saturation efficiency measurement. **a**: T_1_ map. **b**: Saturation efficiency map. **c**: The histogram of the saturation efficiency. High saturation efficiency (0.999±0.012) over all phantoms (T_1_: 300-2000 ms) was achieved. **Table S1.** The average navigator gating efficiency of IMG_1_ and IMG_2_ during the scans of the proposed sequence on the human subjects (*n* = 12). **Figure S3.** Weighted images acquired by the proposed sequence on one subject without and with using fat-suppression (FS) and the corresponding T_1_ maps. **Figure S4.** Image SNR comparison between the proposed sequence and 2D SASHA. The SNR was defined as signal (S) divided by standard deviation of noise (σ), in which the S was calculated as mean signal intensity of septum (red ROI on the weighted images), while σ was calculated from a ROI covering the left ventricle on the noise images. The unshown images (i.e. IMG_3_-IMG_9_) of 2D SASHA had SNR between that of IMG_2_ and IMG_10_. (DOCX 2258 kb)

## Appendix

### Calculation of the number of idle heartbeats for the equilibrium volume

The number of idle heartbeats *n* before the readout of the equilibrium volume is calculated as

A1 $$ n=\left\lceil \frac{\left({\boldsymbol{T}}_{target}-{T}_{trigger}\right)\ast 60}{HR}\right\rceil $$

where *T*_*target*_ is the targeted minimum T_1_ recovery time (e.g. 6 s in this study), *T*_*trigger*_ is the time from the detected R wave to the readout (see Fig. 1). *HR* represents the heart rates. The square bracket represents the operation of rounding up to an integer. Note that because of this rounding up operation, the true recovery time is always larger than the targeted minimum T_1_ recovery time.

## Abbreviations

2D: Two dimensional
2-Param: Two-parameter
3D: Three timensional
3-Param: Three-parameter
AHA: American Heart Association
bSSFP: Balanced steady-state free precession
CV: Coefficient of variation
ECG: Electrocardiogram
ECV: Extracellular volume
FA: Flip angle
FS: Fat saturation
IMG: Image
IR-SE: Inversion-recovery spin-echo
LV: Left ventricle/left ventricular
MOLLI: Modified look-locker inversion recovery
NAV: Navigator
ROI: Region of interest
SASHA: Saturation-recovery single-shot acquisition
SAT: Saturation preparation
SAX: Short-axis
SD: Standard deviation
SNR: Signal-to-noise ratio
SPGR: Spoiled gradient echo
SPIR: Spectral presaturation with inversion recovery
WET: Water suppression enhanced through T_1_ effects
