# Supplementary material for: Three-dimensional free breathing whole heart cardiovascular magnetic resonance T1 mapping at 3 T
Source: J Cardiovasc Magn Reson. 2018 Sep 17;20:64. doi: 10.1186/s12968-018-0487-2 (PMC6139904; doi:10.1186/s12968-018-0487-2)
Supplement: Supplementary file 1 — Figure S1. The mean coefficient of variation of phantom T1 measured by the proposed sequence under different readout flip angles. Figure S2. Results of the phantom experiment for saturation efficiency measurement. a: T1 map. b: Saturation efficiency map. c: The histogram of the saturation efficiency. High saturation efficiency (0.999±0.012) over all phantoms (T1: 300-2000 ms) was achieved. Table S1. The average navigator gating efficiency of IMG1 and IMG2 during the scans of the proposed sequence on the human subjects (n = 12). Figure S3. Weighted images acquired by the proposed sequence on one subject without and with using fat-suppression (FS) and the corresponding T1 maps. Figure S4. Image SNR comparison between the proposed sequence and 2D SASHA. The SNR was defined as signal (S) divided by standard deviation of noise (σ), in which the S was calculated as mean signal intensity of septum (red ROI on the weighted images), while σ was calculated from a ROI covering the left ventricle on the noise images. The unshown images (i.e. IMG3-IMG9) of 2D SASHA had SNR between that of IMG2 and IMG10. (DOCX 2258 kb) [file 12968_2018_487_MOESM1_ESM.docx]

**Supporting Information S1. Coefficient variation of phantom T_1_ measured by the proposed sequence with different flip angles**

Phantom experiments were performed to study the impact of the flip angle of readout of the proposed sequence on the measured T_1_. The proposed sequence were performed with flip angle 3°, 6°, 9°, 12°, 15°, 18°, 21°, 24°, 27°, 30°, 40°, 50° and 60°, respectively. The TR and TE ranged from 2.2 ms to 4.8 ms and 0.73 ms to 1.34 ms, respectively for these scans. The others imaging parameters were the same with the phantom experiment detailed in the manuscript. The mean coefficient variation (CV) of T_1_ of all phantoms under each flip angle was calculated.

The results are shown in Fig. S1. As can be seen, both very low and very high flip angle would cause large CV, while a flip angle of 18° seems to be a good choice as it is far away from both the too low and too high zones.

**
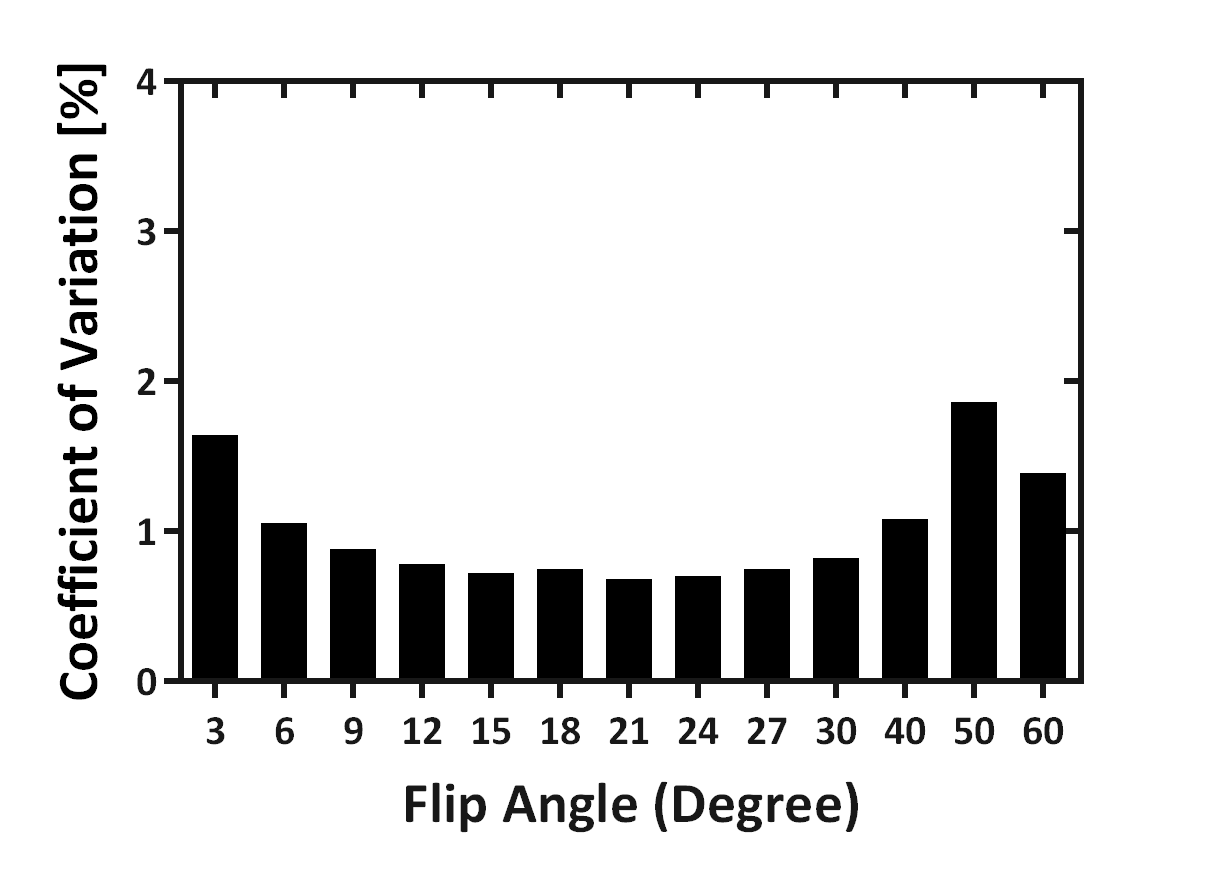
**

**Fig. S1.** The mean coefficient of variation of phantom T_1_ measured by the proposed sequence under different readout flip angles.**Supporting Information S2. Results of the phantom experiment for measuring the saturation efficiency of WET pulse**


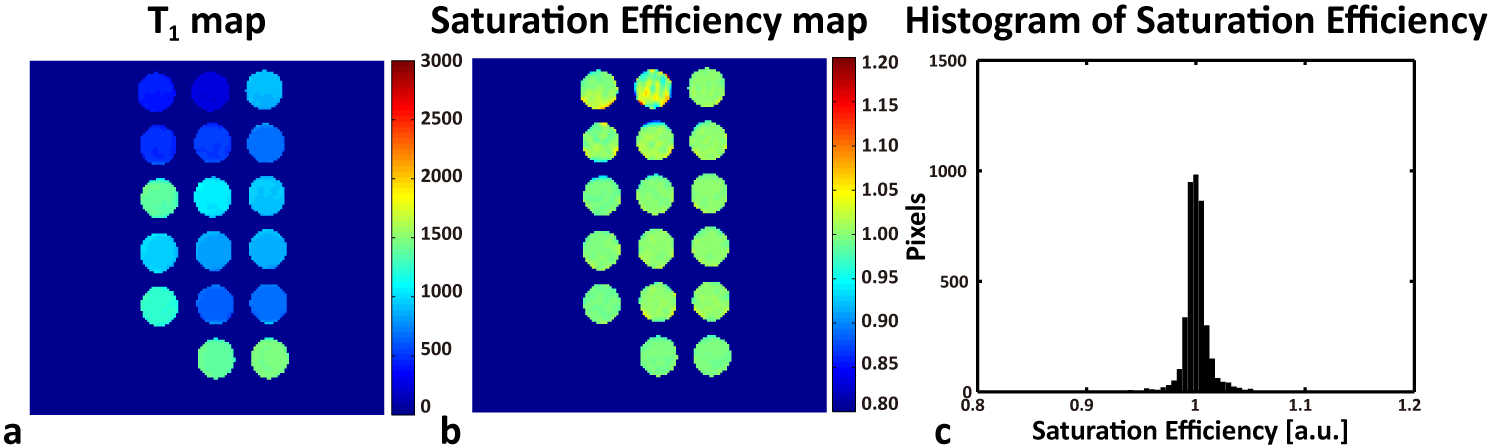


**Fig. S2.** Results of the phantom experiment for saturation efficiency measurement. **a**: T_1_ map. **b**: Saturation efficiency map. **c**: The histogram of the saturation efficiency. High saturation efficiency (0.999±0.012) over all phantoms (T_1_: 300-2000 ms) was achieved.

**Supporting Information S3. The gating efficiency of the navigator for IMG_1_ and IMG_2_**

For the proposed sequence, the free-breathing acquisition is made possible by using navigator gating. Reliability of this navigation depends on the SNR of the navigator signal. In the proposed sequence, the navigator will undergo the same saturation preparation as the heart, and the most questionable navigation is the one for IMG_1_, because it experiences the shortest saturation delay time and thus has the lowest signal SNR.

We analyzed the gating efficiency of the navigator for IMG_1_ and IMG_2_ in the in-vivo experiments. The gating efficiency for the volume was calculated by using the following equation:

$$eff=\frac{1}{N}\sum_{k=1}^{N} \frac{1}{R_{k}+1}$$

where $R_{k}$ is the number of rejected heartbeats before readout of the *k*th shot, *N* is number of shots.

The results for all subjects are shown in Table S1. As can be seen, the gating efficiency for IMG_1_ was not decreased due to its short saturation recovery time.

**Table S1.** The average navigator gating efficiency of IMG_1_ and IMG_2_ during the scans of the proposed sequence on the human subjects (n = 12).

| **Subject** | **HR** | **IMG_1_** | **IMG_2_** |
| --- | --- | --- | --- |
| **1** | 60.4 | 43.2 | 43.2 |
| **2** | 64.0 | 79.3 | 45.1 |
| **3** | 70.6 | 46 | 46 |
| **4** | 72.2 | 29.3 | 25.9 |
| **5** | 76.8 | 100 | 62.5 |
| **6** | 88.2 | 44.4 | 42.1 |
| **7** | 74.1 | 45.6 | 37.5 |
| **8** | 53.8 | 72.7 | 57.1 |
| **9** | 55.0 | 56.4 | 50 |
| **10** | 58.9 | 56.1 | 44.2 |
| **11** | 63.7 | 33.3 | 40 |
| **12** | 69.5 | 82.8 | 85.7 |
| **Mean** |  | 57.4±20.7 | 48.2±14.3 |

**Supporting Information S4. *In vivo* experiments performed with and without using fat-suppression**

The proposed sequence was performed with and without using fat-suppression (FS) technique (i.e. SPIR) on a subject to observe the influence of fat under free-breathing imaging. Other imaging parameters were the same with those detailed in the manuscript. The results are shown in Fig. S3. As can be seen, the T_1_-weighted images acquired without using FS were seriously degraded by the respiratory artifacts arose from fat. These artifacts also decreased the quality of the resulting T_1_ map. Therefore, in this study, the FS was employed in the *in vivo* scans.


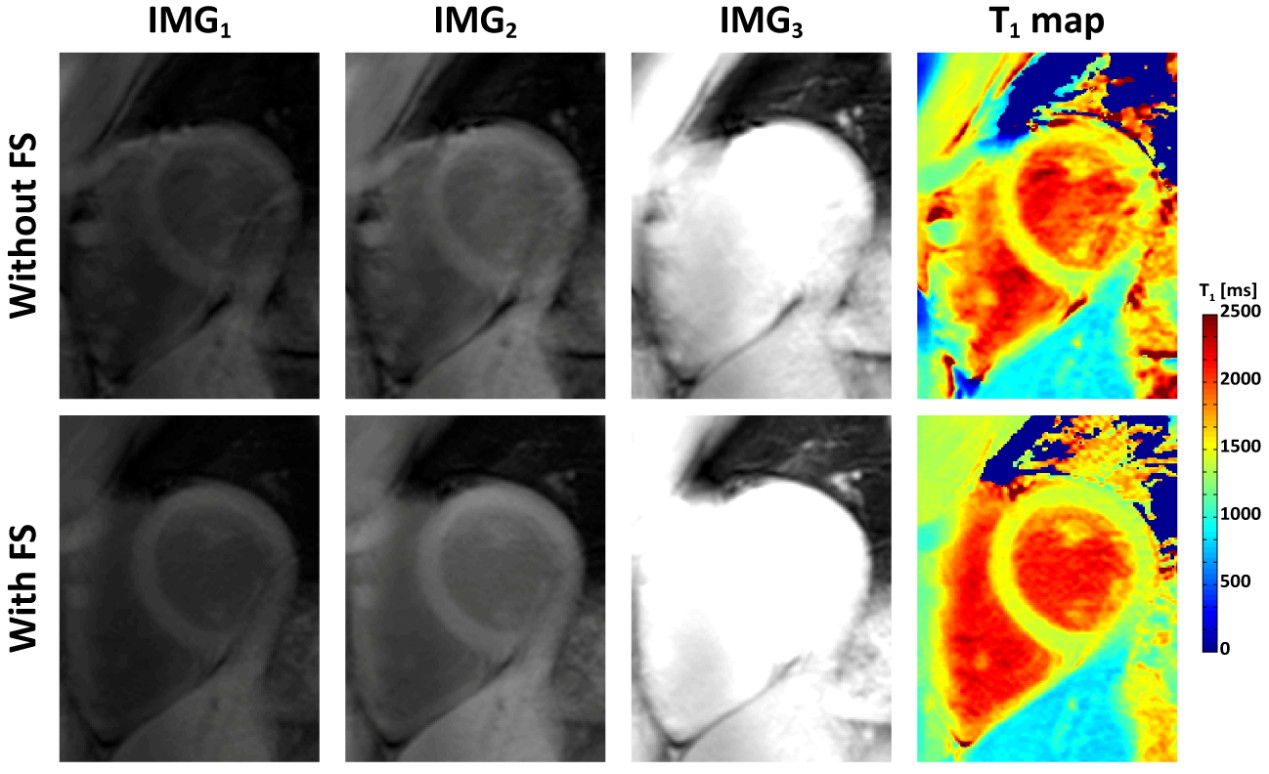


**Fig. S3.** Weighted images acquired by the proposed sequence on one subject without and with using fat-suppression (FS) and the corresponding T_1_ maps.

**Supporting Information S5. Image SNR of the proposed sequence and 2D SASHA**

An additional experiment on two healthy volunteers was performed to compare the image SNR between the proposed sequence and 2D SASHA. Either sequence was scanned twice, one for the normal measurement and the other for noise measurement. The imaging parameters were the same with those detailed in the manuscript except that amplitude of the excitation RF pulse was set to zero in noise measurement. One of the slices acquired by the proposed sequence, which had the same location as the slice acquired by 2D SHSHA, was used to compare with 2D SASHA. Then, to obtain SNR that was defined as signal divided by standard deviation of noise, the signal was calculated as the mean septum signal on the normal raw weighted images, while the standard deviation of noise was obtained from a ROI covering the left ventricle. Fig. S4 shows the drawn ROIs and the resulting SNRs on one of the two volunteers (the other volunteer had similar results). As can be seen, the SNR of the raw weighted images acquired by the proposed sequence was higher than that of 2D SASHA except the first image.

**
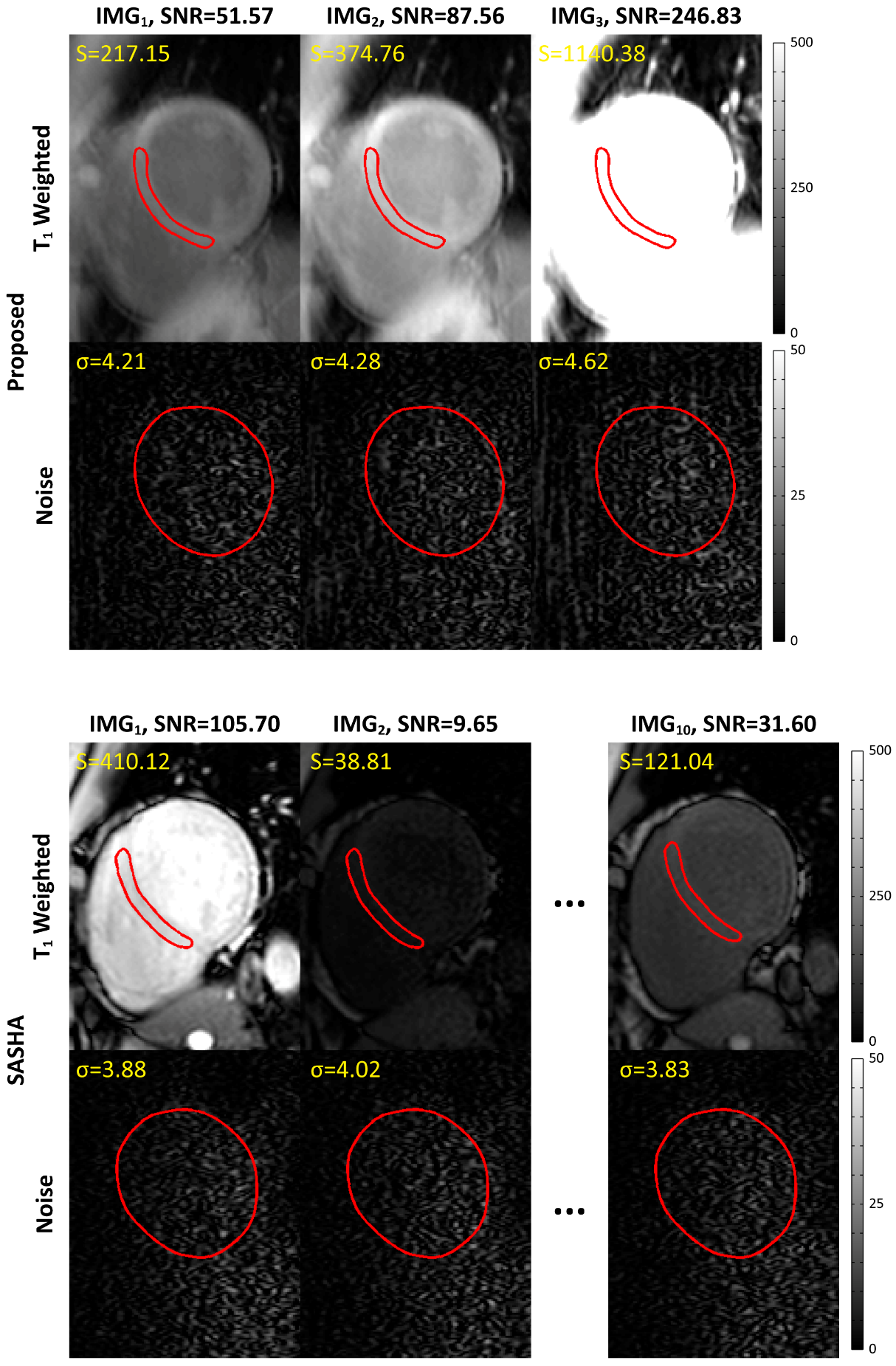
**

**Fig. S4.** Image SNR comparison between the proposed sequence and 2D SASHA. The SNR was defined as signal (S) divided by standard deviation of noise (σ), in which the S was calculated as mean signal intensity of septum (red ROI on the weighted images), while σ was calculated from a ROI covering the left ventricle on the noise images. The unshown images (i.e. IMG_3_-IMG_9_) of 2D SASHA had SNR between that of IMG_2_ and IMG_10_.
